# Novel fusion peptide‐mediated siRNA delivery using self‐assembled nanocomplex

**DOI:** 10.1186/s12951-021-00791-x

**Published:** 2021-02-12

**Authors:** Yeong Chae Ryu, Kyung Ah Kim, Byoung Choul Kim, Hui-Min David Wang, Byeong Hee Hwang

**Affiliations:** 1 Department of Bioengineering and Nano-bioengineering, Incheon National University, Academy-ro 119, Yeonsu-gu, Incheon, 22012 Korea; 2 Division of Bioengineering, Incheon National University, Incheon, 22012 Korea; 3 Graduate Institute of Biomedical Engineering, National Chung Hsing University, Taichung, 402 Taiwan

**Keywords:** Nanocomplex, Self‐assembly, Peptides, siRNA, Drug delivery, Gene silencing

## Abstract

**Background:**

Gene silencing using siRNA can be a new potent strategy to treat many incurable diseases at the genetic level, including cancer and viral infections. Treatments using siRNA essentially requires an efficient and safe method of delivering siRNA into cells while maintaining its stability. Thus, we designed novel synergistic fusion peptides, i.e., SPACE and oligoarginine.

**Results:**

Among the novel fusion peptides and siRNAs, nanocomplexes have enhanced cellular uptake and gene silencing effect in vitro and improved retention and gene silencing effects of siRNAs in vivo. Oligoarginine could attract siRNAs electrostatically to form stable and self-assembled nanocomplexes, and the SPACE peptide could interact with the cellular membrane via hydrogen bonding. Therefore, nanocomplexes using fusion peptides showed improved and evident cellular uptake and gene silencing of glyceraldehyde 3-phosphate dehydrogenase (GAPDH) via the lipid raft-mediated endocytosis pathway, especially to the HDFn cells of the skin, and all of the fusion peptides were biocompatible. Also, intratumorally injected nanocomplexes had increased retention time of siRNAs at the site of the tumor. Finally, nanocomplexes demonstrated significant in vivo gene silencing effect without overt tissue damage and immune cell infiltration.

**Conclusions:**

The new nanocomplex strategy could become a safe and efficient platform for the delivery of siRNAs into cells and tissues to treat various target diseases through gene silencing.
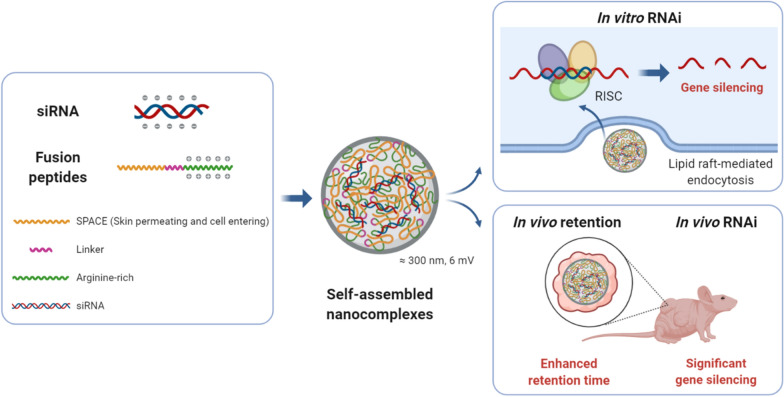

## Background

RNA interference (RNAi) has been demonstrated to be a promising gene silencing approach that regulates the expression of specific genes [1–3]. As an RNAi mediator, short-interfering RNA (siRNA) is a double-stranded molecule that is composed of about 21–23 nucleotides, and it is designed as a sequence complementary to the target mRNA. The exogenously penetrated siRNAs activate the RNA-induced silencing complexes (RISC) in the cytoplasm and result in selective mRNA inhibition with low cytotoxicity. Therefore, gene silencing using siRNA can be a new potent strategy to treat cancer, viral infectious diseases, and local diseases at the genetic level. However, a significant barrier to siRNA delivery is that its hydrophilic nature results in low uptake efficiency into the cell membranes that are composed of phospholipid bilayers [4, 5]. Also, the siRNA is vulnerable to degradation by the large amounts of nucleases that are present in the cytoplasm or interstitial fluid [6]. Therefore, it is essential to develop an efficient, safe, and stable method of delivering siRNA.

To date, several methods have been developed to deliver siRNA, and they can be categorized as either physical or chemical methods [7]. The physical methods could deliver siRNAs using specialized equipment, e.g., microinjectors, gene guns, electroporators, sonoporators, lasers, and magnetofectors [8]. However, physical methods have limitations for various applications due to the need for special equipment, non-specificity of the delivery, and the instability of siRNA that is delivered. Also, chemical methods could deliver siRNAs by using carriers that are capable of interacting with them and transferring them into cells. The potential types of carriers include conjugated and unconjugated forms of lipoplex, polyplex, dendrimer, peptide, and various nanoparticles [9–13]. As the RNAi therapeutics, FDA-approved ONPATTRO^®^ and GIVLAARI™ have the delivery carrier of lipid nanoparticles and GalNAc-siRNA conjugates, respectively. These delivery carriers have been widely applied to RNAi therapeutics in phases 2 and 3 of clinical trials [14, 15]. These carriers could enhance the stability of siRNAs and the efficiency of delivery. However, the chemical methods have the disadvantages of limited delivery efficiency, additional conjugation, or the potential toxicity of the chemicals. Therefore, an ideal siRNA delivery method requires enhanced delivery efficiency, biosafety, and siRNA stability.

Recently, peptides have been studied intensively as attractive siRNA carriers due to their structural and functional versatility, potential biocompatibility, and their ability to target cells. Primarily, cell-penetrating peptides (CPPs) have been known to penetrate cell membranes effectively. The TAT sequence originated from the Tat protein of the human immunodeficiency virus (HIV) [16–18]. Oligoarginine is positively charged, and it can assist cellular internalization by forming a hydrogen bond with the sulfate of the cell membrane and the phosphate group of nucleic acid [19–23]. The histidine-rich peptide was confirmed using the efficient delivery of siRNA [24]. In addition, the development of the phage display technique made it possible for us to find new types of cell-penetrating peptides. For example, the skin permeating and cell entering (SPACE) peptides have a superior ability to facilitate the penetration of conjugated cargoes into the epidermis and dermis [25]. However, limited delivery efficiency was observed for a single peptide, and some peptides such as SPACE, must undergo the additional conjugation reaction. Therefore, for a facile and useful siRNA carrier, a method is required that provides enhanced delivery efficiency without further reaction.

**Fig. 1:**
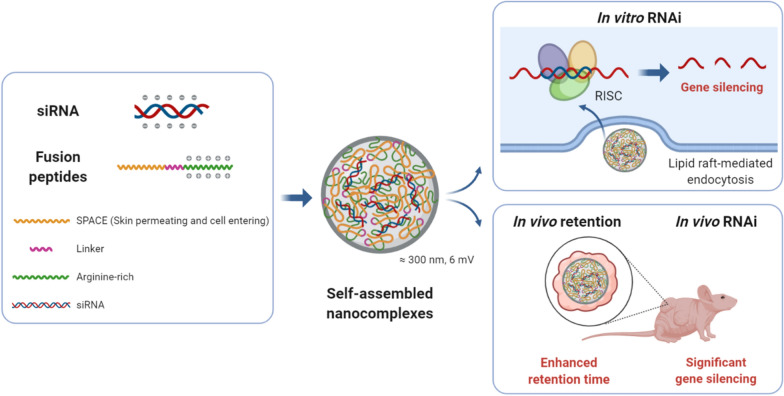
Scheme of in vitro and in vivo delivery using siRNA/fusion peptide nanocomplexes. The nanocomplex could be self-assembled via electrostatic attraction between the negatively-charged siRNA and positively-charged arginine-rich region of the fusion peptides, as well as hydrophobic interaction between amphipathic SPACE regions of fusion peptides. The hydrophilic part of the SPACE peptide might have been exposed to the surfaces of the nanocomplexes. The nanocomplex had a diameter of 300 nm and 6-mV zetapotential with a slight positive charge, and it efficiently penetrated the cellular membrane via lipid raft-mediated endocytosis pathway. The siRNAs were released from the nanocomplex in cells, bound to RISC, and mediated effective gene silencing of specific mRNA. The intratumorally administered nanocomplex enhanced retention time of siRNAs at the site of the tumor on the mouse. And, the subcutaneously-injected nanocomplexes with transiently mCherry-expressing cells showed significant in vivo gene silencing effect. The figure was created using BioRender (https://biorender.com)

Herein, we report our design of novel fusion peptides and the results of our investigation of their potential as carriers for the delivery of siRNA (Fig. 1). The three fusion peptides were composed of SPACE and cationic oligoarginine (R7, R11, and R15) linked by the GCG sequence (Additional file 1: Table S1) [26]. The self-assembled nanocomplex was identified between each peptide and siRNA without any conjugation. Also, each nanocomplex was characterized in terms of size, zeta potential, and siRNA stability. The cellular uptake efficiency of each nanocomplex was measured using flow cytometry and fluorescence microscopy. Intracellular co-localization or dissociation of the nanocomplex was analyzed using a confocal microscope. The nanocomplex-mediated GAPDH knockdown was assessed through the mRNA expression level. And, the biocompatibility of each nanocomplex was checked using a lactate dehydrogenase assay of human dermal fibroblast cells. Also, the internalization pathway of the siRNA/S-R15 nanocomplex was analyzed using endocytosis inhibitors and flow cytometry. Finally, the pharmacokinetic property of the Cy3-labeled siRNA/S-R11 nanocomplex was studied using intratumoral injection to xenografted BALB/c nude mice. The pharmacodynamic property of siRNA was assessed using the subcutaneous injection of the nanocomplex-applied cells to BALB/c nude mice. The potential safety of nanocomplex was explored using histological analysis of intradermally nanocomplex-injected mice skin tissues stained by hematoxylin and eosin (H&E).

## Results

Novel fusion peptides were designed using SPACE and oligoarginine with different repeat numbers for siRNA delivery through self-assembled nanocomplexes. The newly-synthesized fusion peptides successfully formed stable and spontaneous nanocomplexes with siRNAs mainly via electrostatic attraction. All of the nanocomplexes enhanced the cellular uptake of siRNAs such that it was similar to or better than commercialized Lipofectamine™ 2000. Co-localization and cellular internalization of the siRNA/S-R15 nanocomplexes were verified peripherally around the nucleus. Among fusion peptides, the S-R15 nanocomplex induced the highest knockdown of GAPDH mRNA expression, i.e., it was comparable to that of commercialized Lipofectamine™ 2000. Also, each fusion peptide was biocompatible with human dermal fibroblast cells at a concentration of 200 µg/mL. The primary penetration mechanism of the S-R15 nanocomplex was identified as lipid raft-mediated endocytosis. In xenografted BALB/c nude mice, the nanocomplex stabilized and kept the locally administered siRNAs in the tumor site. In addition, nanocomplex-mediated siRNA delivery enhanced in vivo gene silencing effect than naked siRNA delivery. Finally, the nanocomplex in this study did not explicitly damage the tissues or induce immune cell infiltration.

### Confirmation and characterization of siRNA/peptide nanocomplexes

**Fig. 2:**
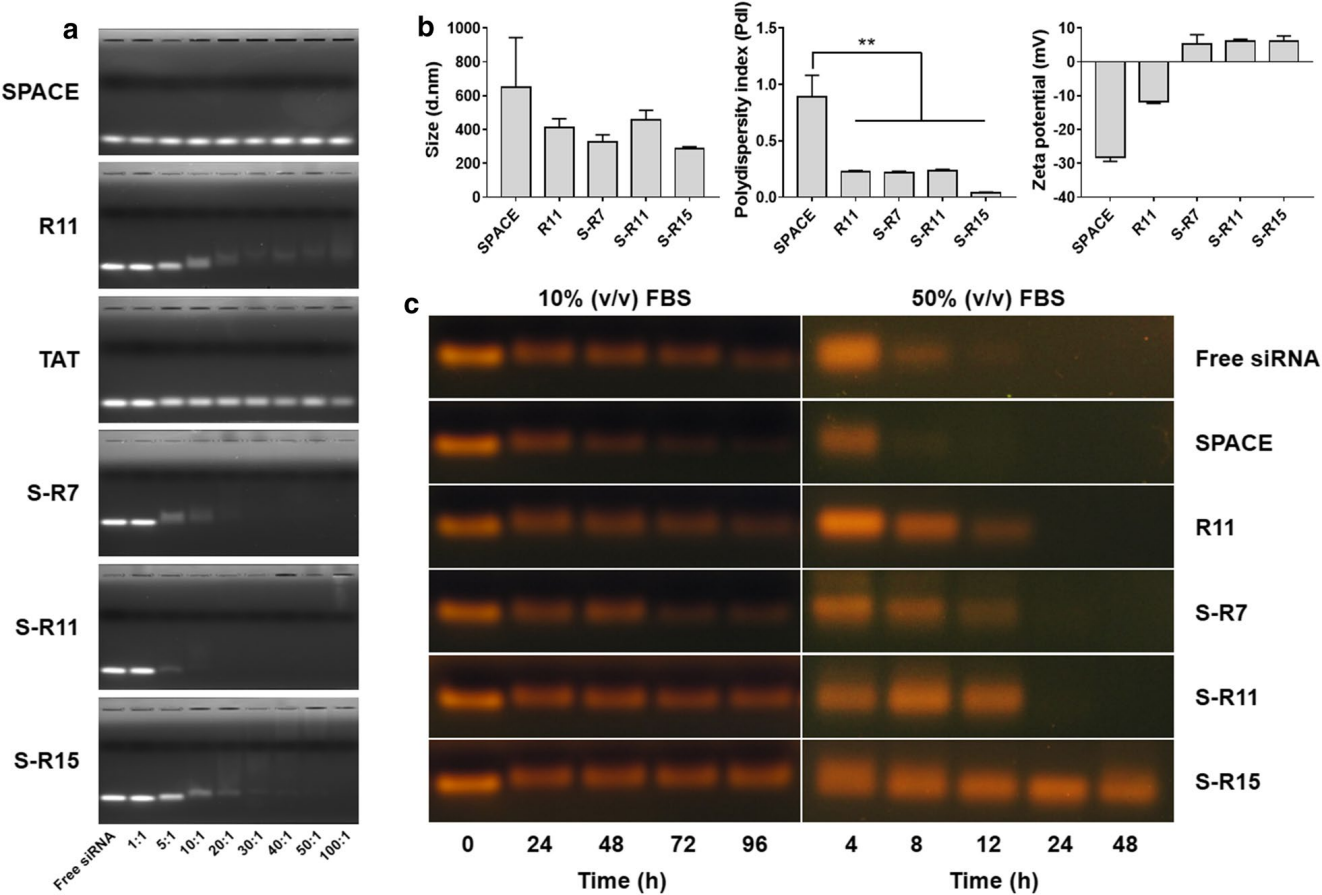
Characterization of siRNA/peptide self-assembled nanocomplexes: **a** Nanocomplex formation was checked using a gel retardation assay. The 21-bp siRNA was mixed with each peptide, i.e., SPACE, R11, TAT, S-R7, S-R11, and S-R15 at N/P ratios of 1:1, 5:1, 10:1, 20:1, 30:1, 40:1, 50:1, and 100:1. After incubation for 30 min, nanocomplexes mixed with a 6× loading dye were loaded into 2% (w/v) agarose gel stained with TopRed. Gel electrophoresis was run in TAE buffer at 100 V for 30 min. The gel was visualized through a ChemiDoc™ XRS + System. The brightness and contrast of each picture were adjusted. **b** The sizes and zeta potentials of the nanocomplexes were measured using dynamic light scattering. The siRNA of 200 nM final concentration was incubated for 30 min with each peptide: SPACE, R11, S-R7, S-R11, and S-R15. After filtration and vortexing, each nanocomplex was loaded in the cell and analyzed through Nano ZS. Bars represented the average ± standard deviation. The *p*-value was calculated using a t-test compared to that of SPACE (**p < 0.01, independent n = 3). **c** siRNA stability was tested in serum. In the left pictures, each siRNA/peptide nanocomplex was incubated in 10% FBS for 24, 48, 72, and 96 h. In the right images, each nanocomplex was incubated in 50% FBS for 4, 8, 12, 24, and 48 h. Then, the samples were mixed with a 6× loading dye and loaded into a 2% (w/v) agarose gel stained with TopRed. Gel electrophoresis was run in the TAE buffer at 100 V for 25–30 min. The gel was visualized through a gel documentation system. The brightness and contrast of each picture were adjusted for the best visualization

The formation of siRNA/peptide nanocomplexes at different N/P ratios was confirmed by electrophoretic mobility shift (Fig. 2a). As a result, R11, S-R7, S-R11, and S-R15 retarded the siRNA band. As fusion peptides, S-R7, S-R11, and S-R15 showed complete retardation of siRNA at ratios over 20:1, 10:1, and 40:1, respectively. Partial retardation was observed with R11, a single peptide, based on the blur siRNA band at ratios over 30:1. However, SPACE and TAT did not retard the siRNA band at all for any of the N/P ratios. Because TAT, a single peptide, did not form a condensed nanocomplex with siRNA, peptides other than the TAT peptide were used for the following experiments.

The size and zeta potential of the nanocomplex were measured three times using dynamic light scattering (Fig. 2b and Additional file 1: Table S2). Nanocomplexes using SPACE, R11, S-R7, S-R11, and S-R15 had average sizes of 648, 414, 327, 457, and 287 nm in hydrodynamic radius, respectively. Nanocomplexes that used SPACE, R11, S-R7, S-R11, and S-R15 had average polydispersity indexes (PdI) of 0.89, 0.23, 0.22, 0.23, and 0.04, respectively, and they had average zeta potentials of − 28.3, − 11.8, 5.2, 6.0, and 6.1, respectively.

The stability of siRNA in nanocomplexes was assessed during the incubation of the serum (Fig. 2c). First, each nanocomplex was incubated with 10% FBS to simulate a cell culture condition. Interestingly, the nanocomplexes that used S-R15 and S-R11 maintained the siRNA stability for 96 h. However, the siRNA bands of the other nanocomplexes disappeared gradually over time. The decomposition rates in 10% FBS increased in the order of S-R15, S-R11, R11, free siRNA, S-R7, and SPACE. Subsequently, each nanocomplex was incubated with 50% FBS to simulate extreme decomposition conditions. Interestingly, only the S-R15 nanocomplex maintained siRNA stability for 48 h, and the siRNA bands of the other nanocomplexes disappeared gradually, and completely in 24 h. The decomposition rates in 50% FBS increased in the order of S-R15, S-R11, R11, S-R7, free siRNA, and SPACE. S-R15 showed the best siRNA stability in the nanocomplex for both conditions.

### Evaluation of the in vitro cellular uptake of the nanocomplexes

**Fig. 3:**
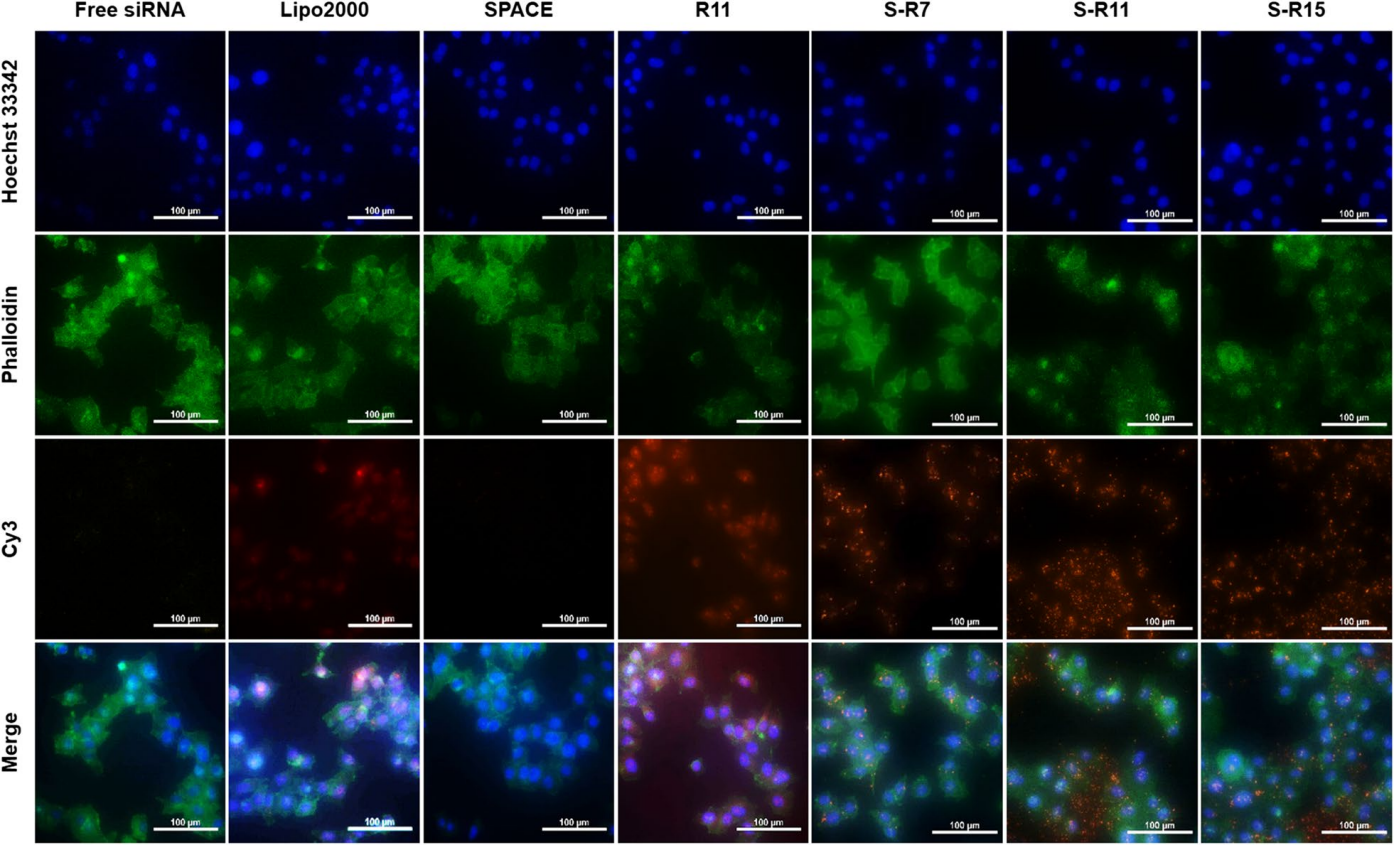
Evaluation of the cellular uptake of siRNA/peptide nanocomplexes using a fluorescence microscope. 200 nM Cy3-labeled siRNAs were delivered into 1.0 × 10^5^ HeLa cells via PC: Lipofectamine™ 2000, SPACE, R11, S-R7, S-R11, and S-R15 (20:1 N/P ratio) for 4 h. Nucleus and actin filaments were labeled using Hoechst 33342 (blue) and Phalloidin (green), respectively. The nanocomplex was observed in orange at 200× magnification (scale bar = 100 µm)

The cellular uptake of each nanocomplex was observed using a fluorescence microscope in HeLa cells (Fig. 3). The images represented Cy3-labeled siRNA of orange fluorescence, the nucleus of blue fluorescence, and the actin filament of green fluorescence. Fig.ure 3 shows that orange fluorescence was observed inside the cells in the images of Lipofectamine™ 2000 (the second row), R11 (the fourth row), S-R7 (the fifth row), S-R11 (the sixth row), and S-R15 (the seventh row). Nanocomplexes using fusion peptides showed orange spots in the cytosol. However, Lipofectamine™ 2000 showed dispersed orange fluorescence within the cytosol, and an R11 nanocomplex showed orange fluorescence spots that were spread within the cytosol. In contrast, free siRNA and SPACE nanocomplex did not show any orange fluorescence in the first and third rows of Fig. 3.

**Fig. 4:**
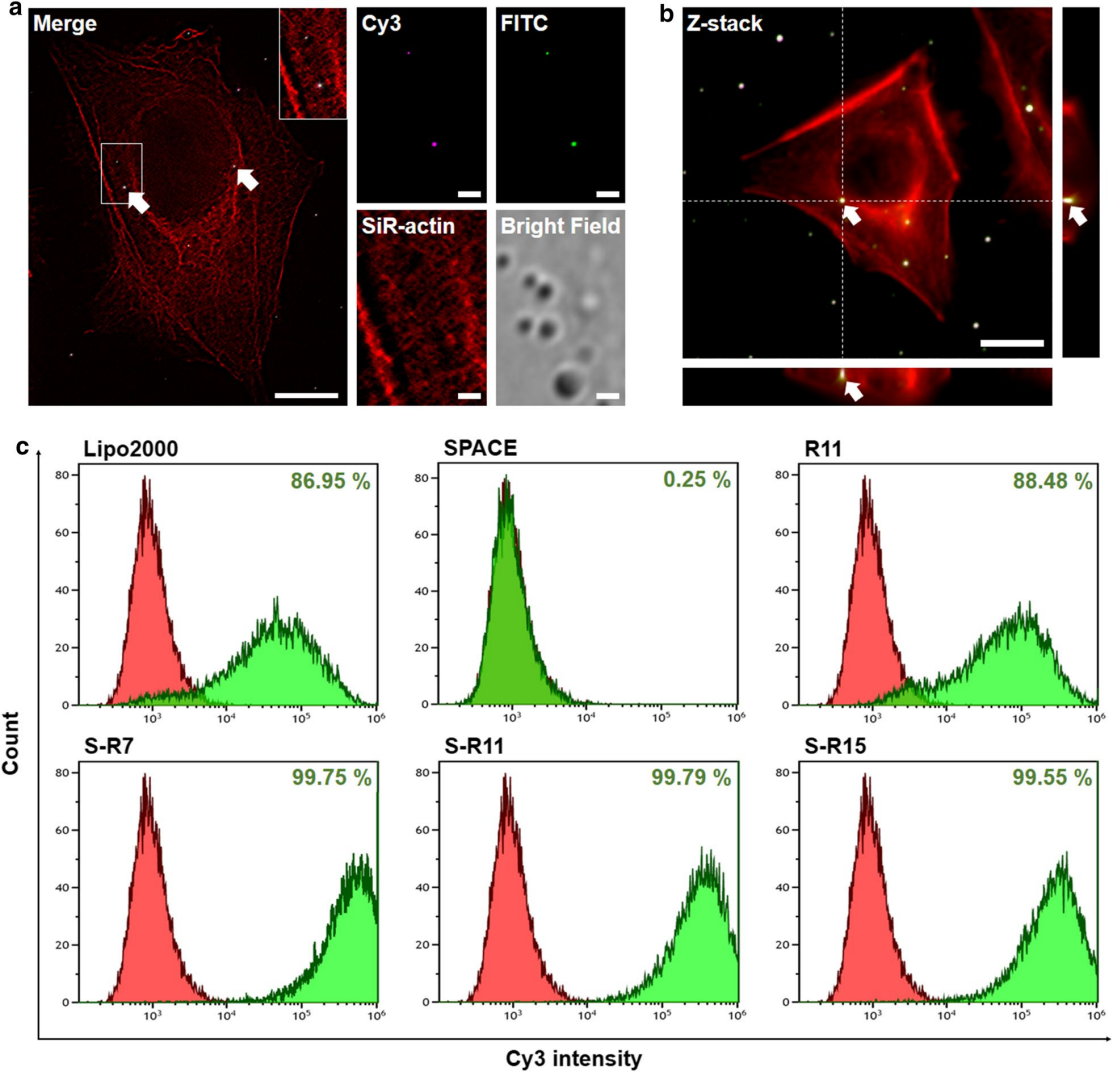
Evaluation of the cellular uptake of siRNA/peptide nanocomplexes using fluorescence analysis: **a** Single-molecule images of the siRNA/S-R15 nanocomplex were acquired using a super-resolution radial fluctuation. 2.0 × 10^4^ HeLa cells were incubated in a 35-mm confocal dish. The nanocomplex with the final 50 nM of siRNA and S-R15 (20:1 N/P ratio) was applied to the cells for 4 h. Cy3-labeled siRNA and FITC-labeled S-R15 peptide were observed in magenta and green, respectively, at 900× magnification (scale bar = 1 µm). Actin filaments were labeled using a SiR-actin kit (red). Fluorescence images of Cy3, FITC, and SiR-actin were merged using ImageJ software. Co-localization of the nanocomplex was visualized with arrowed white spot-like areas in a merged image (scale bar = 10 µm). **b** Cellular internalization of the siRNA/S-R15 nanocomplex was confirmed using a Z-stack image. Actin filaments were labeled using a SiR-actin kit (red). The arrowed white spot-like areas demonstrated co-localization of siRNA and peptide in the cytoplasm at 900× magnification (scale bar = 5 µm). The right and bottom images showed a cross-sectional z-axis image of the arrowed white spot. **c** Cellular uptake of the Cy3-labeled siRNA/peptide nanocomplexes was evaluated using flow cytometry. 200 nM siRNAs were delivered into 3.0 × 10^5^ HDFn cells via PC: Lipofectamine™ 2000, SPACE, R11, S-R7, S-R11, and S-R15 (20:1 N/P ratio) for 4 h. Fluorescence cells of free siRNA and each condition were exhibited in red and green populations, respectively. The population of fluorescence-positive cells was expressed as a percentage.

Also, co-localization and cellular internalization of nanocomplexes were confirmed using a confocal microscope with super-resolution at the single-molecule level (Fig. 4a, b). The Cy3-modified siRNA and FITC-modified S-R15 peptide in the images were represented as magenta fluorescence and green fluorescence, respectively (Fig. 4a). The siRNA and S-R15 peptides were co-localized at the white spot designated by the arrow in the merged image. The nanocomplexes of the Cy3-labeled siRNA and FITC-labeled S-R15 were localized intracellularly in the Z-stack image of HeLa cells (Fig. 4b). The arrows point to the spots where white fluorescence was located in the cytoplasm around the nucleus.

The cellular uptake efficiency of each nanocomplex was evaluated using flow cytometry in HDFn (Fig. 4c), HeLa, and HaCaT cells (Additional file 1: Fig. S1). Using Cy3-labeled siRNA, the fluorescent cells with free siRNA and each condition were exhibited in red and green populations, respectively. The percentage represented the population of fluorescence-positive cells divided by the total cells. Nanocomplexes using S-R7, S-R11, and S-R15 showed the high cellular uptake efficiencies of 99.8%, 99.8%, and 99.6% in the HDFn cells (Fig. 4c), 95.6%, 85.2%, and 78.2% in the HeLa cells, and 95.5%, 79.1%, and 99.9% in the HaCaT cells (Additional file 1:  Fig. S1). These efficiencies were higher than 87.0% in HDFn cells, 71.3% in HeLa cells, and 79.8% in HaCaT cells treated by Lipofectamine™ 2000 as a commercialized positive control. The nanocomplex using R11, a single peptide, showed cellular uptake efficiency of 88.5% in HDFn cells, 92.6% in HeLa cells, and 99.8% in HaCaT cells. In contrast, the nanocomplex using SPACE showed negligible cellular uptake efficiency in HDFn cells (0.3%), HeLa cells (4.5%), and HaCaT cells (0.2%).

### In vitro gene silencing effect

**Fig. 5:**
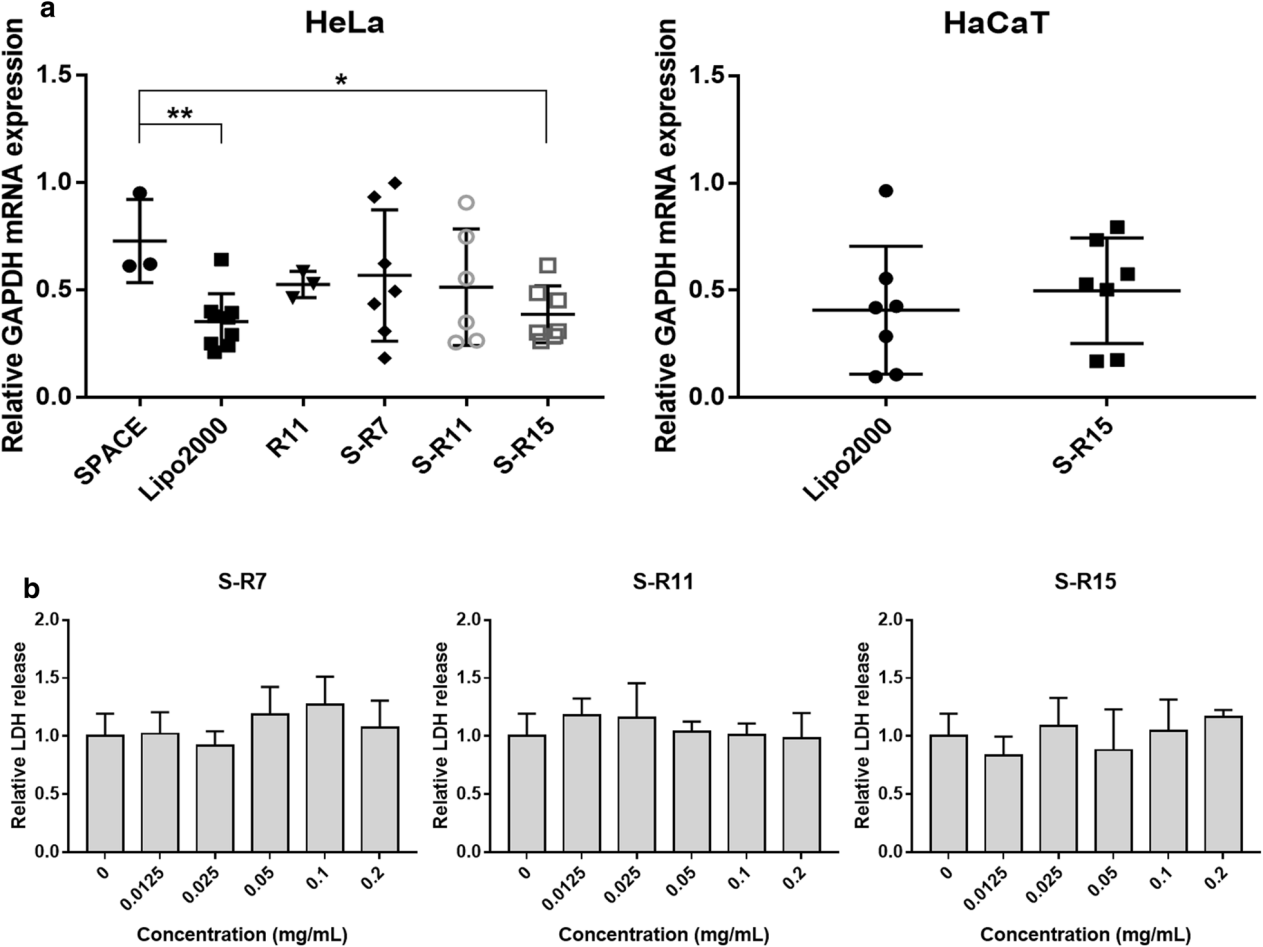
Gene silencing activity and cell viability of fusion peptides: **a** Target GAPDH mRNA knockdown in HeLa and HaCaT by siRNA/peptide nanocomplexes was verified using quantitative RT-PCR. GAPDH-siRNAs of the final 200 nM concentration were delivered into each of the 1.0 × 10^5^ HeLa cells in a 24-well plate using Lipofectamine™ 2000 as a commercialized positive control, SPACE, R11, S-R7, S-R11, and S-R15 (20:1 N/P ratio) for 5 h. 100 ng of total RNAs isolated from cells were reverse-transcribed into cDNA. 10 ng of cDNA were used for the PCR reaction with GAPDH-specific forward and reverse primers. Relative mRNA expression levels were calculated using the ΔΔCt method based on the housekeeping β-actin expression level. The relative expression levels of GAPDH mRNA were normalized by the mRNA expression of free siRNA. The data represented mean ± standard deviation (*p < 0.05, **p < 0.01, and independent n ≥ 3). **b** Cell viability of three fusion peptides was examined using a lactate dehydrogenase (LDH) assay. Released LDH activities were measured from 8.0 × 10^3^ HDFn cells under each fusion peptide at different concentrations (0, 0.0125, 0.025, 0.05, 0.1, and 0.2 mg/mL) in a 96-well plate using an LDH assay. LDH activity of cells treated with each peptide was normalized with the LDH activity without the peptide. The data represented mean ± standard deviation (independent n = 3). The *p*-value was calculated using a t-test.

The siRNA nanocomplex-mediated knockdown of the GAPDH mRNA expression in the HeLa and HaCaT cells was analyzed using quantitative RT-PCR (Fig. 5a and Additional file 1: Table S3). In the HeLa cells, the nanocomplex using S-R15 reduced 61.3% of the relative GAPDH mRNA expression compared to that of free siRNA. This knockdown percentage was significantly different from that of SPACE (*p*-value = 0.011) and comparable to 64.7% using Lipofectamine™ 2000. Nanocomplexes using R11, S-R7, and S-R11 downregulated the GAPDH mRNA expression of 47.4, 43.2, and 48.7%, respectively. Also, the nanocomplex using SPACE induced the least knockdown of 27.2%. In the HaCaT cells, the 50.2% knockdown using S-R15 was comparable to 59.2% of Lipofectamine™ 2000 without a statistically significant difference. These results indicated that these nanocomplexes could knock down mRNA expression in different types of cells, including cancer cells and keratinocyte cells in the skin.

### Biocompatibility evaluation of fusion peptides

The biocompatibility of fusion peptides was verified using a lactate dehydrogenase (LDH) assay with HDFn cells (Fig. 5b and Additional file 1: Table S4). Each LDH activity was normalized with the LDH activity using a lysis buffer as 100%. The normalized LDH activity of various concentrations of each fusion peptide did not show any significant cytotoxicity compared to the negative control. Therefore, fusion peptides were deemed to be biocompatible and could be applied to the cells at concentrations of less than 200 µg/mL.

### Cellular uptake pathway of the nanocomplex

**Fig. 6:**
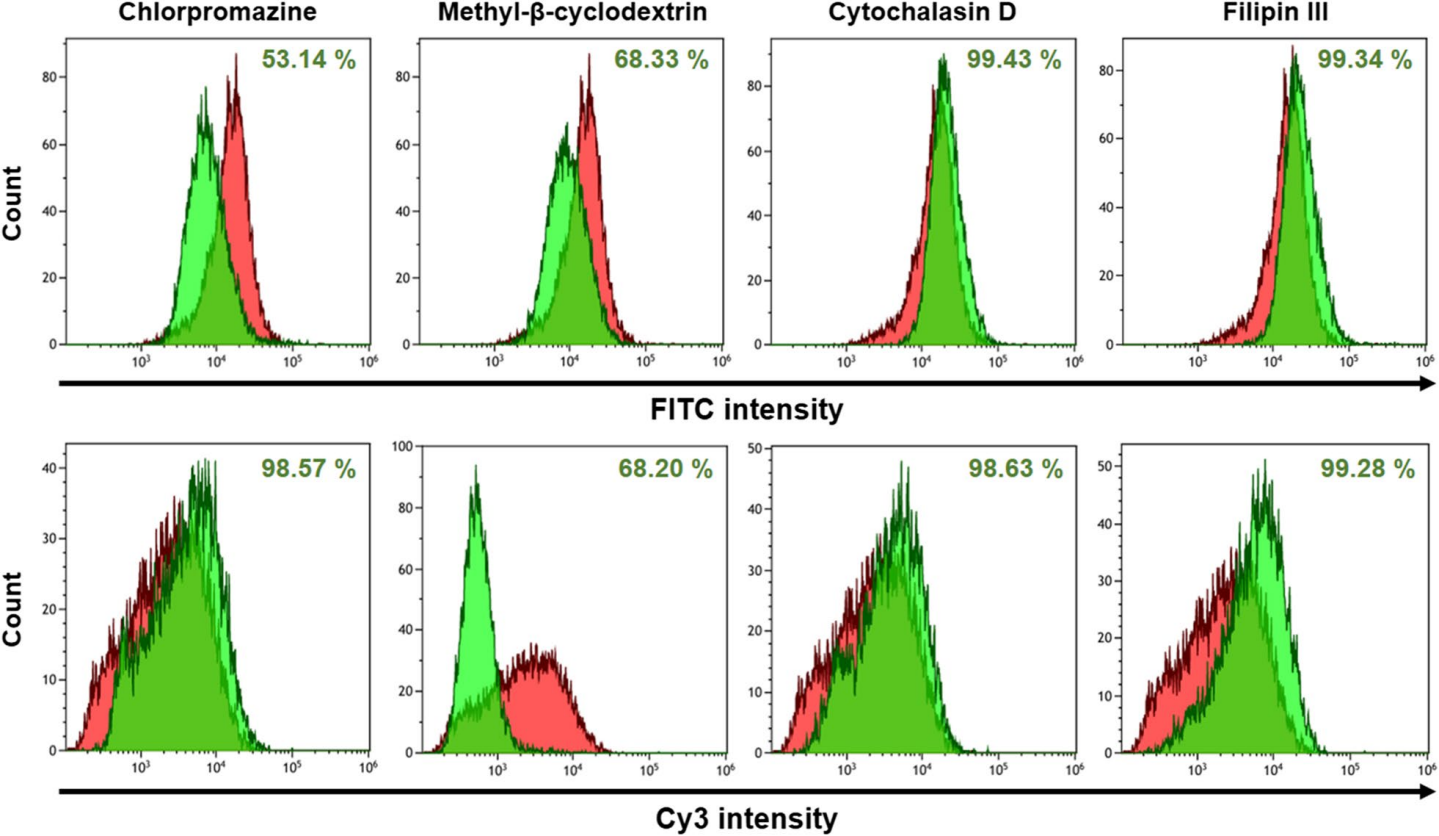
Endocytosis pathway identification of the siRNA/S-R15 nanocomplex using various chemical inhibitors. 4.0 × 10^5^ HeLa cells were pretreated with each endocytosis inhibitor (10 µg/mL of chlorpromazine, 5 mM of methyl-β-cyclodextrin, 1 µM of cytochalasin D, and 1 µg/mL of filipin III) for 30 min. The final 100 nM concentration of the Cy3-labeled siRNA/FITC-labeled S-R15 nanocomplex (20:1 N/P ratio) was delivered into the cells for 4 h. Each fluorescent cell population was counted using detached cells via FITC (upper column) and Cy3 (lower column) intensities, respectively. The fluorescent cell populations without and with each inhibitor were exhibited in red and green, respectively. The reduced population of fluorescence-positive cells was expressed as a percentage

The cellular uptake pathway of the nanocomplex was analyzed using endocytosis inhibitors and flow cytometry (Fig. 6). On the graphs, cell distributions without an inhibitor were represented as red, and those with an inhibitor were represented as green. The figures of the first column only expressed the penetrating inhibition of the FITC-labeled S-R15 peptide or that of the nanocomplex. When the reference point was taken at 89.5% in red distribution, the population of cells decreased to 53.1% in chlorpromazine and to 68.3% in methyl-β-cyclodextrin. Chlorpromazine and methyl-β-cyclodextrin are known as inhibitors of clathrin-meditated endocytosis and lipid raft-mediated endocytosis, respectively. In contrast, the cell populations showed no decrease in the cases of cytochalasin D and filipin III. Cytochalasin D and filipin III are known as inhibitors of phagocytosis/micropinocytosis and caveolae-meditated endocytosis, respectively. Thus, the penetration of the S-R15 peptide only or the nanocomplex was inhibited dominantly by clathrin-mediated and lipid raft-mediated endocytosis.

The figures of the second column represented the penetrating inhibition of the Cy3-labeled siRNA nanocomplex. When the reference point was set at 89.2% in red distribution, the cell population decreased to 68.2% prominently in the lipid raft-mediated endocytosis inhibitor, methyl-β-cyclodextrin. In contrast, the other cell populations with other inhibitors showed no decreases. Therefore, the permeation of the siRNA nanocomplex was inhibited dominantly when lipid raft-mediated endocytosis was inhibited. In summary, both experiments using FITC-labeled S-R15 peptide only or nanocomplex and Cy3-labeled siRNA nanocomplex showed consistency of inhibition when lipid raft-mediated endocytosis was inhibited.

### In vivo siRNA retention effect

**Fig. 7:**
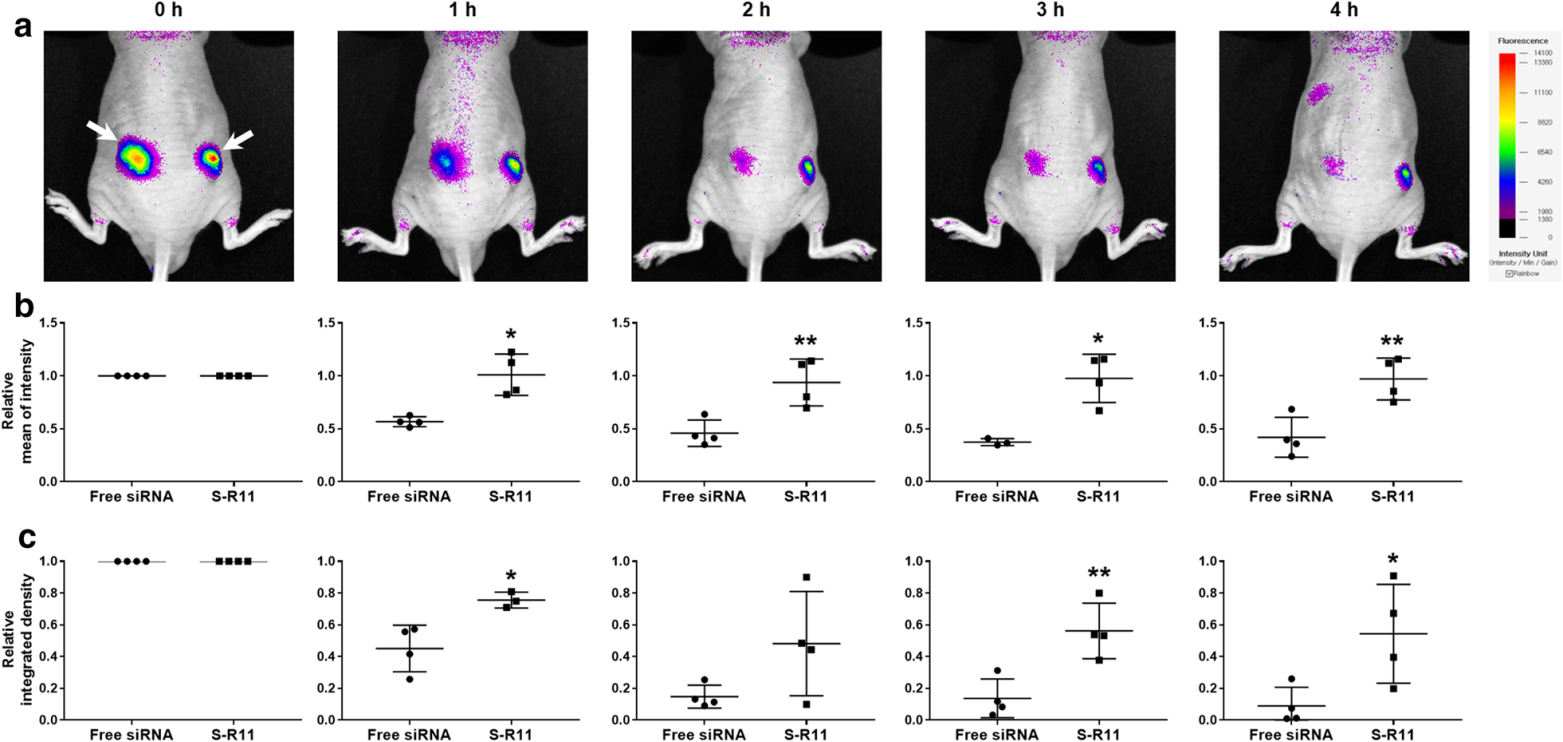
In vivo fluorescence imaging of intratumorally administered siRNAs to tumor-xenografted mice. The BALB/c nude mice were anesthetized with 1.5−2% isoflurane, and 100 µL of 6.0 × 10^6^−1.0 × 10^7^ HeLa cells were inoculated subcutaneously on both sides of the back. After 10 days, 1 µg of free Cy3-labeled siRNA was administered intratumorally into the left tumor, and 1 µg of Cy3-labeled siRNA/S-R11 (20:1 N/P ratio) nanocomplex was administered intratumorally into the right tumor site. **a** The Cy3 fluorescence intensity was observed every hour for 4 h using an in vivo fluorescence imaging system. The left arrow indicated the fluorescence distribution of free siRNA, and the right arrow pointed to that of the S-R11 nanocomplex. **b** Relative mean fluorescence intensity and **c** relative integrated density (area × intensity unit) of each independent sample were analyzed using NEOimage software. The intensity at each time point was normalized with the initial intensity. The data represented mean ± standard deviation (*p < 0.05, **p < 0.01, and independent n = 4)

The pharmacokinetic property of siRNA was assessed using the intratumoral injection of the nanocomplex to xenografted mice. Fluorescence images were taken of the Cy3-labeled siRNA/S-R11 nanocomplex (Fig. 7). Immediately after the injection of siRNA (0 h), the higher fluorescence intensity of Cy3-labeled siRNAs was observed in the core region of the right tumor with the nanocomplex than in the region of free siRNAs without the nanocomplex (Fig. 7a). Also, over time, the fluorescence area and intensity of the siRNAs without the nanocomplex were diminished quickly. In contrast, the fluorescence area and intensity of siRNAs in the nanocomplex decreased gradually and remained in the tumor site for at least 4 h.

The relative mean intensity of the fluorescence of the nanocomplex group did not decrease significantly from the initial value over time. In contrast, the free siRNA group had an approximately 50% reduction in relative mean intensity after 1 h (Fig. 7b and Additional file 1: Table S5). Relative mean fluorescence intensities between the two groups showed statistically significant differences at all points. The *p*-values were 0.017, 0.009, 0.012, and 0.007 for 1, 2, 3, and 4 h, respectively. The relative integrated density was calculated using area multiplied by the fluorescence intensity unit divided by the initial value. The relative integrated density of the nanocomplex group remained about 50% for 4 h, while the free siRNA group simultaneously represented the minimal integrated density (Fig. 7c and Additional file 1: Table S6). The relative integrated density of most conditions showed statistically significant differences between the two groups. The *p*-values were 0.019, 0.132, 0.007, and 0.034 for 1, 2, 3, and 4 h, respectively. To summarize, the nanocomplex enhanced the retention effect of the locally-administered siRNAs.

### In vivo gene silencing effect

**Fig. 8:**
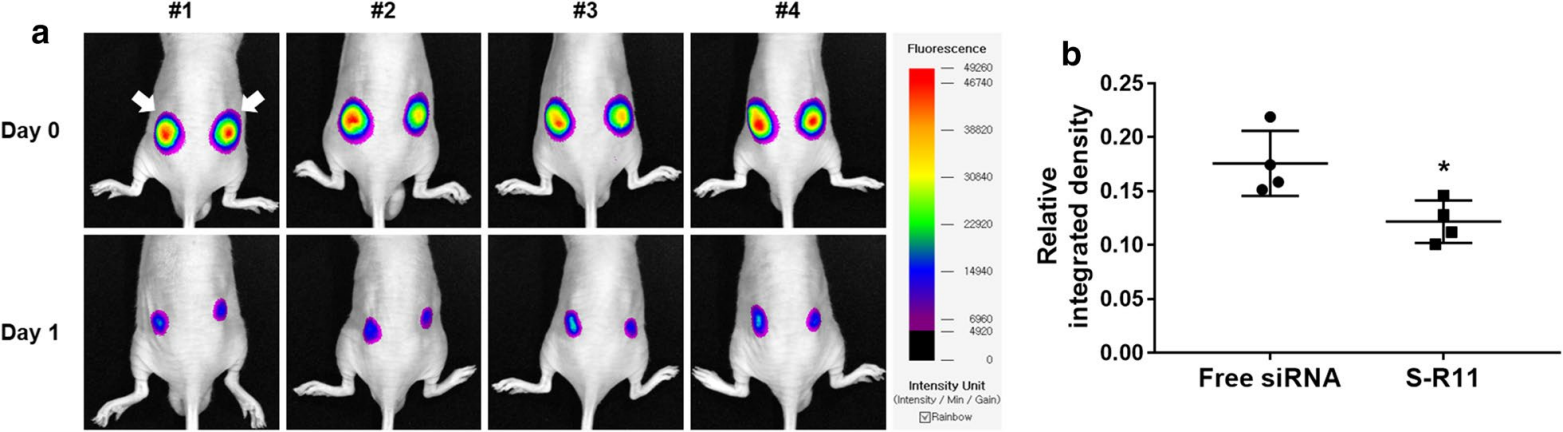
In vivo gene silencing effect using fluorescence imaging. 5.0 × 10^6^ cells of pmCherry-N1 transfected HEK293T with 4 µg of free mCherry-siRNA (left) and siRNA/S-R11 nanocomplex (right) were inoculated subcutaneously on both back sides of the BALB/c nude mice. **a** The mCherry fluorescence intensity was observed on day 0 and day 1 using an in vivo fluorescence imaging system. The left arrow indicated the fluorescence of mCherry-expressing cells with free siRNAs, and the right arrow pointed to that of the S-R11 nanocomplex. **b** Relative integrated density of each mice was analyzed using the NEOimage software. The relative integrated intensity was calculated by that integrated intensity at day 1 was divided by that at day 0. The data represented mean ± standard deviation (*p < 0.05 and independent n = 4)

The pharmacodynamic property of siRNA was assessed using the subcutaneous injection of the nanocomplex-applied cells to BALB/c nude mice. Fluorescence images were taken of the mCherry-expressing cells with free mCherry-siRNA (left) and mCherry-siRNA/S-R11 nanocomplex (right) on day 0 and day 1 (Fig. 8). On day 0, the mCherry fluorescence intensity was uniformly observed on both back sides of the mice (Fig. 8a). On day 1, the fluorescence area and average intensity of the cells applied with nanocomplex (right) were further down compared to free siRNA (left). Also, after the quantitative analysis using an image software, the relative integrated density of the nanocomplex group was significantly lower than that of the free siRNA group (Fig. 8b and Additional file 1: Table S7). The *p*-value was 0.02 by t-test (n = 4). Thus, the nanocomplex improved the in vivo gene silencing effect of the siRNA.

### Histological analysis of skin tissues

**Fig. 9:**
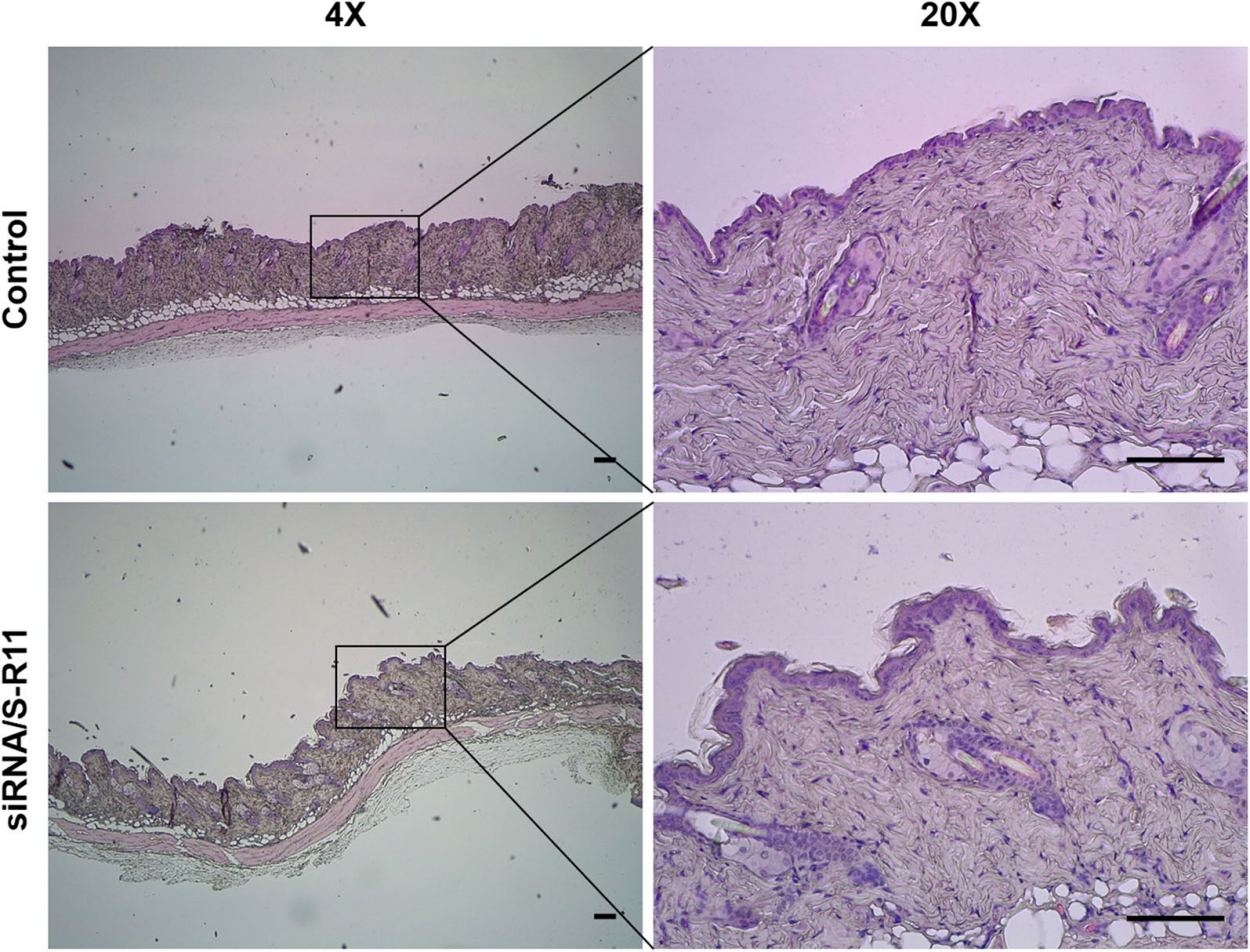
Histological analysis of the nanocomplex-injected skin tissues by hematoxylin and eosin (H&E) staining. The siRNA/S-R11 nanocomplex was injected intradermally to the hairless back of BALB/c mice. After 6 days, the skin tissues were harvested and fixed with 10% neutral buffered formalin. After the standard procedure, the H&E-stained skin tissues were observed using a light microscope under 4× and 20× magnifications (scale bar = 100 µm)

The potential safety of nanocomplex was explored using histological analysis of intradermally nanocomplex-injected mice skin tissues stained by hematoxylin and eosin (H&E). The microscopic images were taken of H&E-stained normal and nanocomplex-treated skin tissues (Fig. 9). The structure of the nanocomplex-treated skin tissue was the same as that of the control group, and no overt tissue damage or immune cell infiltration in the tissue were observed. Consequently, the potential safety of the nanocomplex was confirmed.

## Discussion

Overall, the nanocomplexes that used novel fusion peptides exhibited enhanced cellular uptake, gene silencing effect in vitro and enhanced retention, gene silencing effects of siRNAs in vivo without overt tissue damage. These improved results could be explained based on the intrinsic properties of the nanocomplex, i.e., its uniform nanosize, weakly positive surface charge, stability, endosomal escape assisted by arginine residues, strong adhesion, and safety. It was assumed that the properties of the nanocomplex were caused by the synergistic effects between the oligoarginine, which had a strong positive charge, and the SPACE peptide, which showed effective penetration of the cells [25]. In detail, the oligoarginine of the fusion peptide could attract siRNAs as a driving force to form a nanocomplex. And, as the arginine residues increased, the stability of the nanocomplex increased. Also, it could help to exhibit a weakly positive surface charge and to escape endosomes [27]. In addition, because the SPACE peptide could contain many hydroxyl, sulfhydryl, and amino groups, it could form hydrogen bonds with siRNAs or peptides. Hydrogen bonding of SPACE could enhance the stability of the nanocomplex and interact with the cellular membrane and extracellular matrix (ECM), including keratin [28]. As a result, the interaction with the cellular membrane and ECM could increase the cellular uptake or the retention at tissue. Therefore, the fusion strategy of oligoarginine and SPACE peptides demonstrated synergistic properties complementary to single peptides.

The interactions between peptides and siRNAs can be explained based on their respective properties. First, R11 and fusion peptides with strong positive charges could form self-assembled and condensed nanocomplexes with siRNAs based on the complete retardation of the siRNA band. In contrast, SPACE and TAT, with their weaker positive charges, could not build a condensed nanocomplex with siRNA based on almost no retardation of the siRNA band. These results coincided with the result using oligoarginine [29], and they indicated that a positively-charged region with high density was essential for the formation of nanocomplexes with siRNAs. Using the amino acid analysis via the peptide 2.0 web (www.peptide2.com), the percentages of positive residues were 100% of R11, 9.1% among the 11 amino acids of SPACE, and 88.9% among the 9 amino acids of TAT. The fusion peptides’ percentages of positive residues were 38.1% of S-R7, 48% of S-R11, and 55.2% of S-R15. The successive and longer positive charge of the peptide could increase the electrostatic attraction with the negatively-charged siRNAs and result in the formation of a stable nanocomplex. Note that SPACE contained a hydrophobic alanine, two cysteines (SH), two threonines (OH), two glycines, a serine (OH), two glutamines (NH_2_), and a histidine (NH). These dominant functional groups could form hydrogen bonds and help to maintain the condensed nanocomplexes.

Characterization of the nanocomplexes could help in understanding the reasons for the enhanced cellular uptake and gene silencing. Interestingly, based on the light-scattering analysis, the S-R15 peptide formed the smallest and most uniform nanocomplex with a weakly positive zeta potential. The S-R7 and S-R11 nanocomplexes had small and medium in size, respectively, with acceptable PdI and weak positive charges. The weak positive charges of the three nanocomplexes meant that the fusion peptides were exposed to the surface of the nanocomplex. Also, a slightly positive charge and uniform size could enhance the cellular internalization of the nanocomplex, as reported earlier [30]. Also, the R11 nanocomplex had a medium size, acceptable PdI, and negative zeta potential. Its negative zeta potential could mean that the R11 peptide did not completely shield the negative charge of the siRNAs on the surface of the nanocomplex. In contrast, the SPACE peptide appeared to fail to form a condensed nanocomplex based on its large size, high PdI, and highly negative zeta potential. Consequently, the strategy of fusion peptides enabled the building of nanocomplexes with the proper nanosize, PdI, and weakly positive zeta potential, which could enhance the cellular uptake.

The stability of the nanocomplex could be explained based on the properties of the peptides with different arginine lengths. S-R15 formed the most stable nanocomplex, which might show increased stability upon cellular uptake. The stability of nanocomplexes with siRNAs could be affected by charge neutralization and cohesive strength caused by the electrostatic attraction between the peptides and the siRNAs [31]. That is, more extended and successive positive residues of S-R15 could attract siRNAs and effectively shield the negative charges of siRNA. In addition, the stability of the SPACE part of the S-R11 nanocomplex was comparable to that of the R11 peptide. This result meant that the SPACE peptide could increase the stability of the nanocomplex via hydrogen bonds with siRNAs or peptides.

Clear evidence has been presented that supports the cellular internalization and co-localization of the nanocomplex. Z-stack images showed cellular internalization based on the fluorescence spot in the middle height of a cell (Fig. 4b). Co-localization of the nanocomplex was identified via white fluorescence merged between the magenta fluorescence of the siRNAs and the green fluorescence of the S-R15 (Fig. 4a). The co-localized siRNA/S-R15 nanocomplexes were distributed peripherally around the nucleus in particle-like forms within the cytoplasm. These forms might represent endosomes that contain nanocomplexes. However, the spread of the magenta fluorescence might indicate that the siRNAs dissociated from the nanocomplexes after the endosomal escape. These results coincided with the co-localization or dissociation of nanocomplexes, as reported earlier [29, 32–34].

The efficiency of the cellular uptake of nanocomplexes was different depending on the cell lines. Interestingly, nanocomplexes with fusion peptides showed the highest cellular internalization to human dermal fibroblast cells, i.e., more than 99% (Fig. 4c). These efficiencies were higher than those of the Lipofectamine™ 2000 or the R11 nanocomplex. Similarly, in the HeLa and HaCaT cells, cellular uptakes of fusion peptide nanocomplexes were similar to or higher than that of Lipofectamine™ 2000 (Additional file 1: Fig. S1). In contrast, cellular uptakes of the fusion peptide nanocomplexes were similar to or lower than that of the R11 nanocomplex. As a result, nanocomplexes using fusion peptides could be taken up more efficiently by skin cell lines. These results could be explained in that the SPACE peptide could effectively penetrate skin cells [25].

The endocytosis pathway of the S-R15 peptide does not agree with the micropinocytosis of the SPACE peptide [25], but it does agree with the clathrin-mediated endocytosis of arginine-rich peptides [35]. This result indicated that the S-R15 peptide dominantly could bind the receptors of the surface of a specific cell, resulting in the clustering formed by the assembly of clathrin [35]. However, the endocytosis pathway of the siRNA/S-R15 nanocomplex was different from that of only the S-R15 peptide. The siRNA/S-R15 nanocomplex was associated with the cell membrane and became trapped in lipid raft. The possible reasons could be the large size of the S-R15 nanocomplex and the fact that the strong interactions between oligoarginine and siRNA weakened the binding to the receptors on the surfaces of specific cells. This result coincides with the endocytosis pathway of arginine-rich peptide fusion proteins or large cargo [36].

## Conclusions

Nanocomplexes, among novel fusion peptides and siRNAs, exhibited enhanced cellular uptake, gene silencing effect in vitro and enhanced retention, gene silencing effects of siRNAs in vivo without overt tissue damage. These improved results could be explained by the synergistic effect between the oligoarginine and SPACE peptides. Oligoarginine could electrostatically attract siRNAs to form nanocomplexes, and the SPACE peptide could interact with the cellular membrane via hydrogen bonding. Therefore, nanocomplexes using fusion peptides showed improved and evident cellular uptake and gene silencing of GAPDH via the lipid raft-mediated endocytosis pathway, especially to skin HDFn cells. In addition, all of the fusion peptides were biocompatible. Also, intratumorally injected nanocomplexes showed an increased retention time of siRNAs in the local tumor site. Finally, the nanocomplexes enhanced in vivo target gene silencing effect and validated as a delivery carrier without explicit tissue damage or immune cell infiltration. Therefore, the new nanocomplex strategy could become a safe and efficient siRNA delivery platform to treat various target diseases through gene silencing.

## Methods

### Materials

AccuTarget™ GAPDH positive control siRNA and mCherry-siRNA (sense: 5′-GAGGAUAACAUGGCCAUCAUU-3′, antisense: 5′-UGAUGGCCAUGUUAUCCUCUU-3′) were provided by Bioneer Co. (Daejeon, South Korea), and Cy3-labeled IL10-siRNA (sense: 5′-GCGACGCUGUCAUCGAUUUUU-3′, antisense: 5′-AAAUCGAUGACAGCGUCGCUU-3′) was synthesized by GenePharma Co., Ltd. (Shanghai, China) in duplex form. All siRNAs were purified by HPLC. All single and fusion peptides were synthesized by GL Biochem, Ltd. (Shanghai, China) with more than 95% purity. Hank’s balanced salt solution (HBSS) was obtained from Life Technologies (CA, USA). Agarose and 10,000× TopRed Nucleic Acid Gel Stain were purchased from GenomicBase (Seoul, South Korea). Tris (Glentham Life Sciences Ltd., Corsham, UK), acetic acid (glacial) (Merck, Hesse, Germany), and EDTA (GenomicBase, Seoul, South Korea) were used for the 1× TAE buffer. Heparin sodium salt (from porcine intestinal mucosa) was purchased from Sigma-Aldrich (MO, USA). 6× DNA loading dye was procured from Biofact Co., Ltd. (Daejeon, South Korea). For cell cultures, Dulbecco’s Modified Eagle’s Medium (DMEM; Corning, MA, USA), fetal bovine serum (FBS; PAN Biotech, Bavaria, Germany), and penicillin-streptomycin (Life Technologies, CA, USA) were used. Opti-MEM™ and 0.25% trypsin-EDTA (1×) were obtained from Thermo Fisher Scientific (MA, USA). Lipofectamine™ 2000 reagent was purchased from Invitrogen (CA, USA). Hoechst 33342 (Invitrogen, CA, USA), Flamma^®^ 496 Phalloidin (BioActs, Incheon, South Korea), and SiR-actin kit (Cytoskeleton, Inc., CO, USA) were used for fluorescent labeling. 10% neutral buffered formalin solution (Sigma-Aldrich, MO, USA), Triton X-100 (Bio-Rad Laboratories, Inc., CA, USA), and bovine serum albumin (BSA; Generay Biotech Co., Ltd., Shanghai, China) were used for fluorescent imaging. Nuclease-free water was purchased from Integrated DNA Technologies, Inc. (IA, USA). Tri-RNA reagent (Favorgen Biotech Co., Kaohsiung, Taiwan), chloroform (Sigma-Aldrich, MO, USA), isopropanol (Molecular biology grade; Fisher Scientific, NH, USA), and absolute ethanol (Molecular biology grade; Fisher Scientific) were used for the extraction of RNA. ReverTra Ace^®^ qPCR RT Master Mix with gDNA Remover kit and THUNDERBIRD^®^ SYBR^®^ qPCR Mix (TOYOBO Co., Ltd., Osaka, Japan) were procured for cDNA synthesis and quantitative real-time PCR. CytoTox 96^®^ Non-radioactive cytotoxicity assay kit was obtained from Promega (WI, USA). Chlorpromazine hydrochloride, methyl-β-cyclodextrin, cytochalasin D (from *Zygosporium mansonii*), and filipin III (from *Streptomyces filipinensis*) endocytosis inhibitors were purchased from Merck (Hesse, Germany) for the mechanism study. The lipofector-EXT reagent was obtained from AptaBio (Yongin, South Korea). All cell culture flasks and plates were purchased from NEST Biotechnology Co., Ltd (Wuxi, China). For in vivo studies, isoflurane (Hana Pharm. Co., Ltd., Hwaseong, South Korea) as an anesthetic and 31-gauge needle insulin syringes (BD, NJ, USA) were used.

### Preparation of siRNA/peptide nanocomplexes

GAPDH-siRNA was dissolved in HBSS as 1 µM, and all peptides were dissolved in HBSS or distilled water at 1–2 mg/mL. The sequences of all peptides are summarized in Additional file 1: Table S1. The fusion peptides designed in this study have a GCG linker between SPACE and the oligoarginine peptides. Fusion peptides and siRNAs in HBSS buffer formed the self-assembled nanocomplexes under incubation at room temperature (25°C) for 30 min with appropriate nitrogen/phosphate (N/P) ratio. The N/P ratio was derived from the molar ratio of amine groups in the cationic peptides to phosphate groups in the RNA.

### Gel retardation assay

The formation of siRNA/peptide nanocomplexes was confirmed by gel retardation assay. Total 10 µL nanocomplexes of 10 pmol siRNA and each peptide were self-assembled with a range of N/P ratios (1:1, 5:1, 10:1, 20:1, 30:1, 40:1, 50:1, and 100:1) as mentioned above. After adding 6× loading dye, the 12 µL nanocomplexes were loaded into the 2% (w/v) agarose gel prepared in 1× TAE buffer (40 mM tris, 20 mM acetic acid, 1 mM EDTA, pH 8.6) with 10,000× TopRed Nucleic Acid Gel Stain for visualization. Gel running was performed at 100 V for 30 min using the Mupid-2plus electrophoresis system (Optima Inc., Tokyo, Japan). Pictures of the electrophoretic mobility shift of the nanocomplexes were taken by the ChemiDoc™ XRS + System (Bio-Rad, CA, USA).

### Size and zeta potential measurement

The size and zeta potential of the nanocomplexes were measured by dynamic light scattering (DLS). Based on the results of the previous gel retardation assay, a 20:1 N/P ratio was determined for the rest of the experiments due to the stable nanocomplex formation of all fusion peptides. 200 pmol siRNA and each peptide were self-assembled at a 20:1 N/P ratio, as described above. After 30 min, the nanocomplexes were diluted with HBSS to a final siRNA concentration of 200 nM. 200 nM was chosen as the optimal siRNA concentration based on GAPDH activity assay (Additional file 1: Fig. S2) and used in subsequent experiments. Then, the 200 nM solution was filtered with a 0.45-µm syringe filter (GVS, Bologna, Italy). After vortexing for 30 s, 1 mL of the nanocomplexes was loaded into a cuvette (Ratiolab, Hesse, Germany) to measure the size and disposable folded capillary cell (Malvern Panalytical, Ltd., Malvern, UK) to measure the zeta potential. The size and zeta potential of the nanocomplexes were measured using a Zetasizer Nano ZS (Malvern Instruments, Ltd., Worcestershire, UK).

### Stability of siRNA in serum

The stability of the siRNA in nanocomplexes was confirmed using agarose gel electrophoresis. 100 pmol of siRNAs and peptides (20:1 N/P ratio) were self-assembled for 30 min at room temperature. Then, the nanocomplexes in 10% (v/v) FBS were incubated at 37°C, and 20 µL of each sample was collected at 24, 48, 72, and 96 h. However, the nanocomplex in 50% (v/v) FBS was incubated at 37°C, and 20 µL samples were collected at 4, 8, 12, 24, and 48 h. The siRNAs were dissociated from the nanocomplexes using incubation at 37°C for 30 min after the addition of 4 µL of 1 mg/mL heparin. After mixing the 6× loading dye to each sample, 24 µL samples were loaded into 2% (w/v) agarose gel with 1× TAE buffer in Mupid-2plus. Gel running was performed for 30 min at 100 V. The remaining siRNA was analyzed by the gel documentation system LSG 1000 (iNtRON Biotechnology, Seongnam, South Korea).

### Cellular uptake efficiency using flow cytometry

Human cervical cancer HeLa, human dermal fibroblasts neonatal (HDFn), and immortal keratinocyte cell line HaCaT were cultured in DMEM medium supplemented with 10% FBS and 1% penicillin–streptomycin at 37°C in a humidified incubator (Esco Micro Pte. Ltd., Changi, Singapore) that contained 5% CO_2_. 3.0 × 10^5^ cells were added to each well of a 6-well plate and incubated at 37°C in a 5% CO_2_ incubator overnight. After the nanocomplex formation of the final 200 nM Cy3-labeled IL10-siRNA and peptides (20:1 N/P ratio) in serum-reduced Opti-MEM™, 325 µL of the nanocomplex were added to each well and incubated for 4 h at 37°C in a 5% CO_2_ incubator. The cells were washed twice with 1 mL of pre-warmed phosphate-buffered saline (PBS). After 200 µL treatment of 0.25% trypsin-EDTA for 2 min, 2 mL of fresh DMEM medium was added. The suspended cells were centrifuged at 360×*g* for 5 min. After the supernatant was removed, the cells were washed with PBS twice under the same conditions. The final cell pellets were resuspended in ice-cold PBS and analyzed using a flow cytometer (Gallios; Beckman Coulter, CA, USA).

### Observation of cellular uptake using fluorescence microscopy

HeLa cells were seeded into a 24-well plate at the number of 1.0 × 10^5^ cells per well and incubated overnight at 37°C in a 5% CO_2_ incubator. After the nanocomplex formation of the final 200 nM Cy3-labeled IL10-siRNA and peptides (20:1 N/P ratio), 160 µL of the nanocomplexes were applied to the cells in Opti-MEM™ for 4 h. After washing twice with 200 µL of pre-warmed PBS, the cells were fixed using 200 µL of 10% formalin solution for 10 min. Then, the cells were treated serially with 200 µL of 0.1% Triton X-100 in PBS (0.1% PBST) for 10 min and 200 µL of 2% (w/v) BSA in 0.1% PBST at room temperature for 30 min. The cells were incubated in the Hoechst 33342 dye solution for 10 min in the absence of light. After washing twice with 200 µL of pre-warmed PBS, the cells were incubated in the Phalloidin dye solution at room temperature for 1 h. After washing twice with 200 µL of pre-warmed PBS, the cells were observed at 200× magnification by a fluorescence microscope (Ti-E; Nikon, Tokyo, Japan).

### Cellular internalization observed using confocal microscopy

The cellular internalization of the siRNA/peptide nanocomplex was investigated using confocal imaging. HeLa cells of 2.0 × 10^4^ were incubated in a 35-mm confocal dish (SPL Life Sciences Co., Ltd., Pocheon, South Korea) for 24 h. After a 30-min incubation of the final 50 nM Cy3-labeled siRNA and fluorescein (FITC)-labeled S-R15 (20:1 N/P ratio), the nanocomplex was applied to the cells for 4 h. The nucleus and actin were stained using 5 µg/mL Hoechst 33342 and 100 nM SiR-actin kit, respectively. The intracellular localization and co-localization of siRNA and S-R15 were confirmed using fluorescence and a confocal microscope (Ti2; Nikon, Tokyo, Japan). Both of them were analyzed at the single-molecule level using super-resolution radial fluctuation (SRRF). Bright-field and fluorescence images were acquired at 900× magnification. ImageJ software was used to merge the fluorescence images of Cy3, FITC, and SiR-actin [37].

### Gene knockdown evaluation by quantitative RT-PCR

GAPDH mRNA expression reduced by nanocomplex was checked using quantitative real-time PCR. HeLa cells were seeded into a 24-well plate at a density of 1.0 × 10^5^ cells per well. After overnight incubation at 37°C in a 5% CO_2_ incubator, 160 µL nanocomplex with the final 200 nM siRNA at 20:1 N/P ratio was applied to the cells in Opti-MEM™ for 5 h. As a positive control, Lipofectamine™ 2000 reagent was used according to the provided protocols. After incubation for 5 h, the media were replaced with 500 µL of fresh supplemented DMEM and incubated for an additional 19 h. After washing three times with nuclease-free water, the total RNA of the cells was purified using Tri-RNA reagent following the manufacturer’s protocols. The concentration and purity of purified RNA were measured using the Take3 plate as a nanodrop mode of a microplate reader (Epoch 2; BioTek Instruments, Inc., VT, USA). 100 ng of RNA were transcribed reversely to cDNA using ReverTra Ace^®^ qPCR RT Master Mix with a gDNA Remover kit according to the manufacturer’s protocols. After mixing 10 ng of cDNA, primers, and THUNDERBIRD^®^ SYBR^®^ qPCR Mix according to the manufacturer’s protocol, the PCR reaction was performed following the three-step cycle (Pre-denaturation; 95°C for 60 s, Denaturation; 95°C for 15 s, Annealing; 55°C for 30 s, Extension; 72°C for 60 s). The used primer sequences of GAPDH and β-actin are provided in Additional file 1: Table S3. GAPDH mRNA expression was analyzed using the QuantStudio 3 real-time PCR system (Applied Biosystems, CA, USA). The GAPDH mRNA expression was normalized by β-actin mRNA expression. The relative expression level was calculated using the ΔΔCt method.

### Endocytosis pathway study

In a 6-well plate, HeLa cells were seeded at a density of 4.0 × 10^5^ cells per well. After replacing the medium with Opti-MEM™, the cells were treated with each inhibitor for 30 min, i.e., Chlorpromazine (10 µg/mL), methyl-β-cyclodextrin (5 mM), cytochalasin D (1 µM), and filipin III (1 µg/mL) [38]. The nanocomplex of the final 200 nM Cy3-labeled siRNA and FITC-labeled S-R15 was self-assembled as described above. After adding 325 µL of nanocomplex to the inhibitor-treated cells, the cells were incubated for 4 h at 37°C in a 5% CO_2_ incubator. Cells with the fluorescence of Cy3 and FITC were analyzed using flow cytometry, as mentioned above.

### Biocompatibility of the fusion peptides

The biocompatibility of the fusion peptides was assessed using CytoTox 96^® ^non-radioactive cytotoxicity assay according to the manufacturer’s protocols. Human dermal fibroblasts neonatal (HDFn) cells were cultured in DMEM medium supplemented with 10% FBS and 1% penicillin-streptomycin at 37°C in a 5% CO_2_ humidified incubator. HDFn cells were seeded on a 96-well plate at a density of 8.0 × 10^3^ cells per well. After incubation overnight, serially-diluted fusion peptides were added to cells at concentrations of 1, 0.5, 0.25, 0.125, and 0.0625 mg/mL. After incubation for 5 h, 50 µL aliquots of each well were transferred to each empty well of a 96-well plate. Then, 50 µL of CytoTox 96^®^ reagent was added to each well. Cells were incubated for 30 min at room temperature in light-free conditions. After adding 50 µL of stop solution, absorbance was measured at 490 nm using a microplate reader.

### In vivo siRNA retention effect

All animal experiments were performed according to the protocol approved by the Institutional Animal Care and Use Committee (IACUC) of Incheon National University (INU-ANIM-2020-01). HeLa cells cultured over 90% confluency were prepared to 6.0 × 10^6^−1.0 × 10^7^ cells in 100 µL of fresh DMEM (w/o FBS). Five-week-old BALB/c nude mice (Orientbio, Inc., Seongnam, South Korea) were anesthetized with 1.5–2% isoflurane in pure oxygen gas. Then, the cells were injected subcutaneously into both sides of the backs of the mice with a 1 mL insulin syringe. The formation of massive tumors was confirmed after 10 days. On day 10, the tumor-xenografted mice were anesthetized, and 29.78 µL of 1 µg free Cy3-labeled siRNA was injected intratumorally into the left tumor of a mouse. Then, 29.78 µL of 1 µg Cy3-labeled siRNA in the S-R11 nanocomplex (20:1 N/P ratio, as mentioned above) was injected intratumorally into the right tumor. The fluorescence intensity of Cy3 was visualized every hour up to 4 h using an in vivo fluorescence imaging system (FOBI; CELLGENTEK Co., Ltd., Cheongju, South Korea). The area, mean of intensity, and integrated density (area × mean of intensity) were quantified from the fluorescence images using NEOimage software.

### In vivo gene silencing effect

In vivo gene silencing effect of nanocomplex was assessed using mCherry fluorescence imaging. For the transient expression of mCherry, 32 µg of the pmCherry-N1 vector was transfected into HEK293T cells cultured in a T175 flask over 80% confluency. As a transfection reagent, Lipofector-EXT was used according to the manufacturer’s protocols. After incubation for 5 h in 20 mL of Opti-MEM™, the cell media were replaced with 40 mL of fresh DMEM, and the cells were cultured in the 5% CO_2_ incubator at 37°C for another 2 days. The pmCherry-N1-transfected cells were harvested and prepared at 5.0 × 10^6^ cells/50 µL. Then, 4 µg of mCherry-siRNA in the S-R11 nanocomplex (20:1 N/P ratio, prepared as mentioned above) was added to the pmCherry-N1 transfected cells. The cells with free siRNAs and cells with the siRNA nanocomplex were injected subcutaneously on the left and right back of BALB/c nude mice under 1.5–2% isoflurane anesthesia, respectively. The fluorescence images of mCherry were taken on days 0 and 1 using an in vivo fluorescence imaging system, and quantitative analysis of four mice images was carried out as previously mentioned. The relative integrated intensity was calculated by that integrated intensity at day 1 was divided by that at day 0.

### Histological analysis of skin tissues

The potential safety was explored using histological analysis of the nanocomplex-injected skin tissues. The siRNA/S-R11 nanocomplex was injected intradermally into three spots of the hairless back of anesthetized five-week-old BALB/c mice. The S-R11 nanocomplex (20:1 N/P ratio) was prepared as mentioned above. After 6 days, the mice were euthanized, and the harvested skin tissues were fixed in 10% neutral buffered formalin. Then, the tissues were dehydrated, paraffinembedded, and sectioned to 4 µm thickness. After the deparaffinization, the tissues were stained with standard hematoxylin and eosin (H&E), and observed using a Leica DM1000 LED microscope (Leica Microsystems, Hesse, Germany) under 4× and 20× magnifications.

### Statistical analysis

The quantitative data were presented as mean ± standard deviation. The statistical significance of the differences was evaluated using a *p*-value less than 0.05, 0.01, and 0.001 calculated by a t-test.

## Supplementary Information

**Additional file 1: Table S1.** List of peptides used in this study. **Table S2.** Average size, polydispersity index (PdI), and zeta potential of complexes with siRNA and each peptide. The data represented the mean ± standard deviation (n = 3). **Table S3.** Primer sequences used in real-time PCR. **Table S4.** Absorbance measurement of released LDH. The data represented the mean ± standard deviation (n = 3). **Table S5.** Relative in vivo fluorescence values by normalized mean of intensity. The data represented the mean ± standard deviation (n = 4). The outliers were removed by the Grubbs’ test. **Table S6.** Relative in vivo fluorescence values by normalized integrated density. The data represented the mean ± standard deviation (n = 4). The outliers were removed by the Grubbs’ test. **Table S7.** Relative in vivo fluorescence values by normalized integrated density. The data represented mean ± standard deviation (n = 4). **Figure S1.** Cellular uptake evaluation of siRNA/peptide nanocomplexes into HeLa and HaCaT cells using flow cytometry. Cy3-labeled IL10-siRNAs of final 200 nM concentration were delivered into each 3.0 × 10^5^ cells in a 6-well plate via PC: Lipofectamine™ 2000 as a commercialized positive control, SPACE, R11, S-R7, S-R11, and S-R15 (20:1 N/P ratio) for 4 h. Fluorescent cells of free siRNA and each condition exhibited in red and green populations, respectively. The population of fluorescent-positive cells was expressed as a percentage. **Figure S2.** Optimization of siRNA concentration by GAPDH activity assay. GAPDH-siRNA/S-R15 nanocomplex was delivered into 3.0 × 10^3^ cells with the final siRNA concentrations of 50, 100, 150, 200, and 250 nM in a 96-well plate for 5 h. After the media replacement, the cells were further incubated for 48 h. (a) The GAPDH activities were measured using a fluorescence spectrophotometer following manufacturer’s protocols. The relative GAPDH activity was represented as the GAPDH activity divided by total protein concentration. (b) Total proteins were quantified using Bicinchoninic acid (BCA) assay. Both graphs ​​were expressed based on the value at 50 nM as 100%. The data represented mean ± standard deviation (*p < 0.05, **p < 0.01 calculated by t-test independent n = 3).

## Data Availability

All data generated or analyzed during this study are included in this published article and its additional information files.

## Abbreviations

GAPDH: Glyceraldehyde 3-phosphate dehydrogenase
RNAi: RNA interference
siRNA: Short-interfering RNA
RISC: RNA-induced silencing complexes
CPPs: Cell-penetrating peptides
TAT: Trans-activator of transcription
SPACE: Skin permeating and cell entering
S-R7: SPACE-R7
S-R11: SPACE-R11
S-R15: SPACE-R15
Lipo2000: Lipofectamine™ 2000
