# Supplementary material for: Novel fusion peptide‐mediated siRNA delivery using self‐assembled nanocomplex
Source: J Nanobiotechnology. 2021 Feb 12;19:44. doi: 10.1186/s12951-021-00791-x (PMC7881583; doi:10.1186/s12951-021-00791-x)
Supplement: Supplementary file 1 — Additional file 1: Table S1. List of peptides used in this study. Table S2. Average size, polydispersity index (PdI), and zeta potential of complexes with siRNA and each peptide. The data represented the mean ± standard deviation (n = 3). Table S3. Primer sequences used in real-time PCR. Table S4. Absorbance measurement of released LDH. The data represented the mean ± standard deviation (n = 3). Table S5. Relative in vivo fluorescence values by normalized mean of intensity. The data represented the mean ± standard deviation (n = 4). The outliers were removed by the Grubbs’ test. Table S6. Relative in vivo fluorescence values by normalized integrated density. The data represented the mean ± standard deviation (n = 4). The outliers were removed by the Grubbs’ test. Table S7. Relative in vivo fluorescence values by normalized integrated density. The data represented mean ± standard deviation (n = 4). Figure S1. Cellular uptake evaluation of siRNA/peptide nanocomplexes into HeLa and HaCaT cells using flow cytometry. Cy3-labeled IL10-siRNAs of final 200 nM concentration were delivered into each 3.0 × 10^5^ cells in a 6-well plate via PC: Lipofectamine™ 2000 as a commercialized positive control, SPACE, R11, S-R7, S-R11, and S-R15 (20:1 N/P ratio) for 4 h. Fluorescent cells of free siRNA and each condition exhibited in red and green populations, respectively. The population of fluorescent-positive cells was expressed as a percentage. Figure S2. Optimization of siRNA concentration by GAPDH activity assay. GAPDH-siRNA/S-R15 nanocomplex was delivered into 3.0 × 10^3^ cells with the final siRNA concentrations of 50, 100, 150, 200, and 250 nM in a 96-well plate for 5 h. After the media replacement, the cells were further incubated for 48 h. (a) The GAPDH activities were measured using a fluorescence spectrophotometer following manufacturer’s protocols. The relative GAPDH activity was represented as the GAPDH activity divided by total protein concentration. (b) Total proteins were q [file 12951_2021_791_MOESM1_ESM.docx]

**Additional information**

**Novel fusion peptide-mediated siRNA delivery using self-assembled nanocomplex**

**Yeong Chae Ryu^1^, Kyungah Kim^1^, Byoung Choul Kim^1,2^, Hui-Min David Wang^3^, Byeong Hee Hwang^1,2,*^**

^1^ Department of Bioengineering and Nano-bioengineering, Incheon National University, Incheon, 22012, Korea

^2^ Division of Bioengineering, Incheon National University, Incheon, 22012, Korea

^3^ Graduate Institute of Biomedical Engineering, National Chung Hsing University, Taichung 402, Taiwan

_____

* Corresponding author.

*Address:* Academy-ro 119, Yeonsu-gu, Incheon, South Korea, 22012

*Phone*: +82-32-835-8834

*Fax*: +82-32-835-2699

*E-mail address:* bhwang@inu.ac.kr

**Table S1.** List of peptides used in this study

| **Name** | **Sequence** | **Origin** | **Reference** |
| --- | --- | --- | --- |
| Skin permeating and cell entering (SPACE) | ACTGSTQHQCG | Artificial | [1] |
| Oligo-arginine (R11) | RRRRRRRRRRR | Artificial | [2] |
| TAT | RKKRRQRRR | HIV-1 | [3] |
| SPACE-R7 (S-R7) | ACTGSTQHQCGGCGRRRRRRR | Artificial | In this study |
| SPACE-R11 (S-R11) | ACTGSTQHQCGGCGRRRRRRRRRRR | Artificial | In this study |
| SPACE-R15 (S-R15) | ACTGSTQHQCGGCGRRRRRRRRRRRRRRR | Artificial | In this study |
| * The underlined sequence indicated a linker between two sequences. | | |  |

**Table S2.** Average size, polydispersity index (PdI), and zeta potential of complexes with siRNA and each peptide. The data represented mean ± standard deviation (n=3).

| **Complex with siRNA** | **Size** | **PdI** | **Zeta potential** |
| --- | --- | --- | --- |
| SPACE | 647.7 ± 295.18 | 0.889 ± 0.192 | -28.33 ± 1.079 |
| R11 | 413.73 ± 50.28 | 0.228 ± 0.009 | -11.77 ± 0.451 |
| S-R7 | 327.1 ± 42.63 | 0.217 ± 0.013 | 5.23 ± 2.796 |
| S-R11 | 456.97 ± 56.79 | 0.235 ± 0.013 | 6 ± 0.684 |
| S-R15 | 286.97 ± 11.42 | 0.04 ± 0.006 | 6.08 ± 1.618 |

**Table S3.** Primer sequences used in real-time PCR

| **Gene** | **Sequence** |
| --- | --- |
| GAPDH | Forward : 5’-GTCTCCTCTGACTTCAACAGCG-3’  Reverse : 5’-ACCACCCTGTTGCTGTAGCCAA-3’ |
| β-actin | Forward : 5’-CACCATTGGCAATGAGCGGTTC-3’  Reverse : 5’-AGGTCTTTGCGGATGTCCACGT-3’ |

**Table S4.** Absorbance measurement of released LDH. The data represented mean ± standard deviation (n=3).

| **Concentration (mg/mL)** | **S-R7** | **S-R11** | **S-R15** |
| --- | --- | --- | --- |
| 0 | 1.000 ± 0.195 | 1.000 ± 0.195 | 1.000 ± 0.195 |
| 0.0125 | 1.020 ± 0.186 | 1.179 ± 0.145 | 0.836 ± 0.159 |
| 0.025 | 0.921 ± 0.122 | 1.157 ± 0.300 | 1.086 ± 0.243 |
| 0.05 | 1.185 ± 0.238 | 1.037 ± 0.089 | 0.879 ± 0.352 |
| 0.1 | 1.270 ± 0.243 | 1.007 ± 0.102 | 1.044 ± 0.273 |
| 0.2 | 1.071 ± 0.235 | 0.983 ± 0.216 | 1.167 ± 0.059 |

**Table S5.** Relative *in vivo* fluorescence values by normalized mean of intensity. The data represented mean ± standard deviation (n=4). The outliers were removed by Grubbs’ test.

| **Group** | **0 h** | **1 h** | **2 h** | **3 h** | **4 h** |
| --- | --- | --- | --- | --- | --- |
| Free siRNA | 1 | 0.568 ± 0.047 | 0.459 ± 0.124 | 0.457 ± 0.166 | 0.421 ± 0.189 |
| S-R11 complex | 1 | 1.011 ± 0.196 | 0.938 ± 0.221 | 0.977 ± 0.228 | 0.972 ± 0.198 |

**Table S6.** Relative *in vivo* fluorescence values by normalized integrated density. The data represented mean ± standard deviation (n=4). The outliers were removed by Grubbs’ test.

| **Group** | **0 h** | **1 h** | **2 h** | **3 h** | **4 h** |
| --- | --- | --- | --- | --- | --- |
| Free siRNA | 1 | 0.451 ± 0.147 | 0.148 ± 0.073 | 0.137 ± 0.123 | 0.089 ± 0.118 |
| S-R11 complex | 1 | 0.756 ± 0.05 | 0.482 ± 0.328 | 0.562 ± 0.175 | 0.544 ± 0.311 |

**Table S7.** Relative *in vivo* fluorescence values by normalized integrated density. The data represented mean ± standard deviation (n=4).

| **Group** | **Day 0** | **Day 1** |
| --- | --- | --- |
| Free siRNA | 1 | 0.176 ± 0.03 |
| S-R11 complex | 1 | 0.122 ± 0.02 |


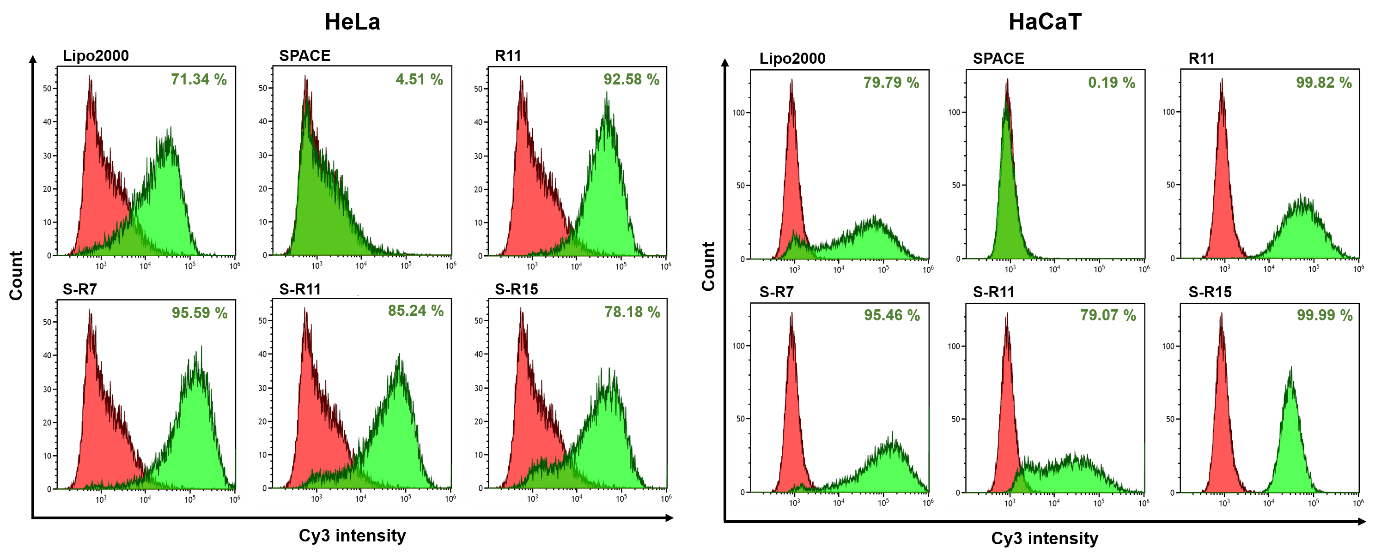


**Figure S1.** Cellular uptake evaluation of siRNA/peptide nanocomplexes into HeLa and HaCaT cells using a flow cytometry. Cy3-labeled IL10-siRNAs of final 200 nM concentration were delivered into each 3.0×10^5^ cells in a 6-well plate via PC: Lipofectamine^TM^ 2000 as a commercialized positive control, SPACE, R11, S-R7, S-R11, and S-R15 (20:1 N/P ratio) for 4 hours. Fluorescent cells of free siRNA and each condition exhibited in red and green populations, respectively. The population of fluorescent-positive cells was expressed as a percentage.


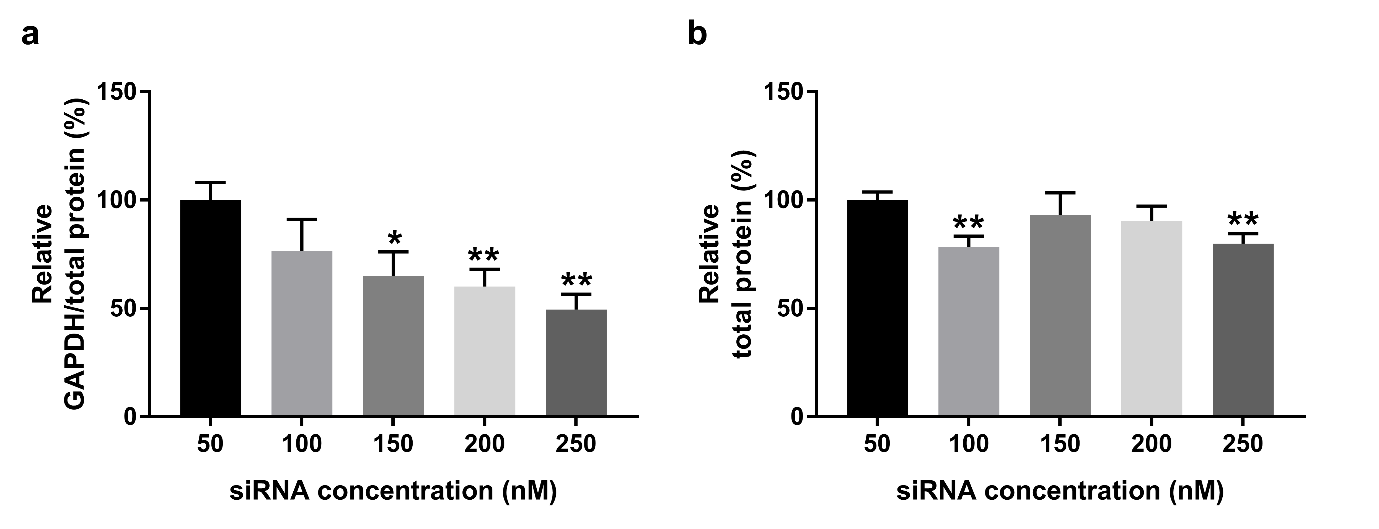


**Figure S2.** Optimization of siRNA concentration by GAPDH activity assay. GAPDH-siRNA/S-R15 nanocomplex was delivered into 3.0×10^3^ cells with the final siRNA concentrations of 50, 100, 150, 200, and 250 nM in a 96-well plate for 5 hours. After the media replacement, the cells were further incubated for 48 hours. (a) The GAPDH activities were measured using a fluorescence spectrophotometer following manufacturer’s protocols. The relative GAPDH activity was represented as the GAPDH activity divided by total protein concentration. (b) Total proteins were quantified using Bicinchoninic acid (BCA) assay. Both graphs ​​were expressed based on the value at 50 nM as 100%. The data represented mean ± standard deviation (*; p < 0.05, **; p < 0.01 calculated by t-test independent n = 3).

**References**

[1] T. Hsu, S. Mitragotri, Delivery of siRNA and other macromolecules into skin and cells using a peptide enhancer, P Natl Acad Sci USA, 108 (2011) 15816-15821.

[2] D.J. Mitchell, D.T. Kim, L. Steinman, C.G. Fathman, J.B. Rothbard, Polyarginine enters cells more efficiently than other polycationic homopolymers, J Pept Res, 56 (2000) 318-325.

[3] E. Vives, P. Brodin, B. Lebleu, A truncated HIV-1 Tat protein basic domain rapidly translocates through the plasma membrane and accumulates in the cell nucleus, J Biol Chem, 272 (1997) 16010-16017.
